# Acute mutism in a young female. A case report of a 20-year-old female who presents a 3-month mutism

**DOI:** 10.1192/j.eurpsy.2023.427

**Published:** 2023-07-19

**Authors:** A. Gonzalez-Mota, A. Gonzalez-Gil, C. Martin-Gomez, J. A. Benito-Sanchez, I. M. Peso-Navarro, L. Fernandez-Alonso

**Affiliations:** ^1^Psychiatry, University Hospital Complex of Salamanca; ^2^Institute of Biomedicine of Salamanca (IBSAL), Salamanca; ^3^Psychology, University Hospital Miguel Servet, Zaragoza; ^4^Psychiatry, School of Medicine, University of Salamanca; ^5^Psychology, University Hospital Complex of Salamanca; ^6^Psychology, School of Medicine, University of Salamanca, Salamanca, Spain

## Abstract

**Introduction:**

A 20-year-old female presents with a progressive 3-month mutism, hyporexia (20kg weight loss), abulia, anhedonia, apathy, social isolation,seeking company of her parents even at night, bradypsychia, sialorrhea, psychomotor slowdown and hypomimia. She is hospitalized in the Psychiatric Brief Hospitalization Unit (PBHU).Her parents relate the beginning of this symptomatology to a breakup and gender violence,which the patient confirms during the interview by eye/cephalic movements and single words jotted down.

**Objectives:**

The objective of this study is to describe the evolution of the patient during her hospitalization in the PBHU of Salamanca and to look into the available bibliography about mutism related to stress and sialorrhea.

**Methods:**

We carried out a follow-up of the hospitalization of the patient and a structured search in PubMed with the keywords “mutism”,“sialorrhea” and “stress” in the last 10 years in English,Spanish and French.

**Results:**

Few or no articles where found.Therefore, the articles about mutism and stress were analyzed, which focused mostly in selective mutism. Regarding fear,the response to cope with the threat(fight, flight, freeze) is mediated by the autonomic system. The “Polyvagal Theory” speaks about the vagus nerve participating in emotion regulation (social communication and mobilization). Dissociation, in this context,has adaptive and defensive purposes and its threshold can be reduced by repeated stress situations.Long-term alteration of the autonomic nervous system has been described in selective mutism.This malfunction can be related to an elevated production of saliva due to the activation of the parasympathetic in the salivary glands, causing sialorrhea in our patient.

The patient began treatment with sertraline 100mg and risperidone 2mg with the aim of its antidepressive and major tranquilizer effects, she also began individual and family psychotherapy, we assured her sleep and intakes and she began to progressively recover her speech and mobility,identifying a possible trigger for the symptomatology: a physical beating of gender violence after her breakup.

**Conclusions:**

Dissociation and “freeze” response can be a maladaptative mechanism to fear.The malfunction of the autonomic nervous system can explain the disconnection,poor gaze,low facial and body expression and inability to speak.

**Disclosure of Interest:**

None Declared

